# Clinical, Functional, and Radiological Profile of Patients With Spondyloarthritis: A Cross-Sectional Study From a Tertiary Care Center in Central India

**DOI:** 10.7759/cureus.116195

**Published:** 2026-09-13

**Authors:** Shilpa S Kuthe, Ajinkya Kakade, Salil Ganu, Fahad Idrees Shaikh, Nishka Tiwari

**Affiliations:** 1 General Medicine, NKP Salve Institute of Medical Sciences & Research Centre and Lata Mangeshkar Hospital, Nagpur, IND; 2 General Medicine, NKP Salve Institute of Medical Sciences & Research Centre, Nagpur, IND; 3 Rheumatology, NKP Salve Institute of Medical Sciences & Research Centre and Lata Mangeshkar Hospital, Nagpur, IND; 4 Medicine, NKP Salve Institute of Medical Sciences & Research Centre and Lata Mangeshkar Hospital, Nagpur, IND; 5 Medical Education, NKP Salve Institute of Medical Sciences & Research Centre and Lata Mangeshkar Hospital, Nagpur, IND

**Keywords:** ankylosing spondylitis, asdas, basdai, basfi, cross-sectional study, hla-b27, spondyloarthritis, vertebral fracture

## Abstract

Background and objectives: Spondyloarthritis (SpA) includes a group of chronic inflammatory rheumatic disorders, such as ankylosing spondylitis, psoriatic arthritis, reactive arthritis, enteropathic arthritis, and undifferentiated SpA, unified by shared genetic, clinical, and immunopathological features. Comprehensive clinical profiling of SpA patients, including disease activity, functional status, and structural complications such as vertebral fracture, remains limited in Indian tertiary care settings. This study aims to describe the sociodemographic, clinical, radiological, and biochemical profile of patients with SpA, including disease activity and functional status using validated indices (Bath Ankylosing Spondylitis Disease Activity Index (BASDAI), Ankylosing Spondylitis Disease Activity Score with C-reactive protein (ASDAS-CRP), and Bath Ankylosing Spondylitis Functional Index (BASFI)), and to assess associations between these measures, inflammatory markers, and the presence of vertebral fractures, a structural complication anticipated to be uncommon in this cohort.

Methods: This hospital-based, cross-sectional, observational study was conducted in the Department of General Medicine of a tertiary care teaching hospital. Fifty adult patients diagnosed with SpA per the European Spondyloarthropathy Study Group (ESSG) criteria were enrolled by consecutive sampling. We performed detailed clinical evaluation, radiological assessment (modified New York criteria for sacroiliitis and vertebral fracture), and laboratory investigations (including HLA-B27 typing). Disease activity and function were assessed using BASDAI, ASDAS-CRP, and BASFI. Data were analyzed using SPSS Statistics version 26.0 (IBM Corp., Armonk, NY, USA); categorical variables were compared using the chi-square/Fisher's exact test and continuous variables using the Student's t-test/Mann-Whitney U test, with p<0.05 considered statistically significant. Given the anticipated small number of vertebral fracture events, all fracture-related comparisons were pre-specified as exploratory.

Results: The cohort had a mean age of 41.8 ± 10.9 years with a male predominance (74%). Ankylosing spondylitis was the most common subtype (54%), and axial involvement was present in 56% of patients; HLA-B27 was positive in 80%. Musculoskeletal (enthesitis, dactylitis) and extra-articular manifestations were noted in 64% of patients overall, most commonly enthesitis (38%) and uveitis (16%). Radiologically, 54% had grade III-IV sacroiliitis, and 12% (n=6) had vertebral fractures (predominantly thoracic, 50%). Fracture prevalence was numerically higher with longer disease duration (p=0.018) and higher erythrocyte sedimentation rate (ESR)/C-reactiive protein (CRP) (p=0.038 and p=0.021, respectively). High disease activity was observed in 72% of patients (BASDAI ≥4.0) and 74% (ASDAS-CRP >2.1); 68% had moderate-to-severe functional limitation (BASFI ≥4.0).

Conclusion: Spondyloarthritis in this cohort predominantly affected young to middle-aged men from rural and lower socioeconomic backgrounds, with a high burden of active disease and structural damage. Vertebral fractures were more frequent with longer disease duration and higher inflammatory markers in this exploratory analysis. The need for early diagnosis, routine disease-activity monitoring, and clinical vigilance for vertebral fracture as an under-recognized structural complication is imperative in resource-limited settings.

## Introduction

Spondyloarthritis (SpA) is a group of chronic inflammatory rheumatic diseases that share overlapping clinical features, genetic predisposition, and immunopathological mechanisms. It encompasses ankylosing spondylitis, psoriatic arthritis, reactive arthritis, enteropathic arthritis, and undifferentiated SpA, collectively characterized by inflammation of the axial skeleton, entheses, peripheral joints, and extra-articular organs such as the eye and gut [1,2]. Historically termed 'seronegative spondyloarthropathies' owing to the absence of rheumatoid factor, the modern Assessment of SpondyloArthritis International Society (ASAS) criteria classify the disease spectrum into axial SpA, with radiographic and non-radiographic subtypes, and peripheral SpA [3-5]. The global prevalence of AS is estimated at 0.1% to 1.4%, with a predilection for young adults and a male preponderance, particularly in radiographic axial disease [6].

The pathogenesis of SpA is multifactorial. The strongest genetic risk factor is posed by HLA-B27, present in 85% to 90% of patients with AS, with additional contributions from dysregulated interleukin-23/interleukin-17 signaling and gut-joint interactions implicated in a subset of patients [7-9]. Clinically, SpA manifests with inflammatory back pain, sacroiliitis, peripheral arthritis, enthesitis, dactylitis, and extra-articular features such as uveitis, psoriasis, and inflammatory bowel disease, producing substantial impairment in physical function, work productivity, and quality of life [10].

Diagnosis of SpA is frequently delayed by several years owing to its insidious and heterogeneous presentation, particularly in non-radiographic disease. Diagnostic delay has been associated with greater structural damage and functional impairment at the time of diagnosis, although this association may be influenced by underlying disease severity, phenotype, and other confounding factors rather than delay in isolation [11]. Musculoskeletal and extra-articular manifestations further complicate the clinical picture and are reported in a substantial proportion of patients across cohorts, adding to overall disease burden [12].

Beyond axial and peripheral joint disease, SpA is increasingly recognized as a disorder of impaired bone health. Chronic systemic inflammation promotes osteoclast-mediated bone resorption, while concurrent syndesmophyte formation and spinal ankylosis paradoxically increase structural rigidity; this combination of an osteoporotic yet biomechanically inflexible spine renders patients with longstanding SpA particularly vulnerable to vertebral fracture, often after minimal or no trauma. Reduced spinal mobility and altered posture may further predispose to falls and abnormal load distribution along the vertebral column. Vertebral fractures in SpA carry disproportionate clinical significance, as they may precipitate neurological injury and worsen pain and disability, yet are frequently under-recognized on routine radiographic assessment.

While the ASAS criteria represent the current standard for classifying axial and peripheral SpA [4,5], the present study enrolled patients using the broader European Spondyloarthropathy Study Group (ESSG) criteria [13], which encompass a wider spectrum of axial, peripheral, and juvenile-onset disease and thereby permit description of a heterogeneous, real-world SpA population attending a general medicine department where full ASAS phenotyping infrastructure (e.g., dedicated sacroiliac joint MRI) is not routinely available. Specifically, the extent to which validated disease activity (Bath Ankylosing Spondylitis Disease Activity Index (BASDAI) [14], Ankylosing Spondylitis Disease Activity Score with C-reactive protein (ASDAS-CRP) [15]) and Bath Ankylosing Spondylitis Functional Index (BASFI) [16] indices relate to the presence of vertebral fracture in Indian patients with SpA has not been well characterized, and comprehensive tertiary-care data linking sociodemographic profile, disease subtype, radiological severity, and structural bone complications remain scarce. This cross-sectional study was therefore undertaken to systematically describe the sociodemographic, clinical, radiological, and biochemical profile of patients with SpA attending a tertiary care hospital and to exploratorily examine associations between disease activity, functional status, and the presence of vertebral fractures.

## Materials and methods

Study design and setting

This is a hospital-based, observational, cross-sectional study conducted in the Department of General Medicine of a tertiary care teaching hospital serving both rural and urban populations in Central India. The Institutional Ethics Committee of NKP Salve Institute of Medical Sciences & Research Centre and Lata Mangeshkar Hospital (Nagpur, MH, IND) issued approval (approval no. 36/2024 dated 18 April 2024). Patients were recruited from the medicine outpatient department (OPD), inpatient department (IPD), and ICU. The study was conducted over a period of 18 months, from 1 May 2024 to 31 December 2025, and was conducted in accordance with the Declaration of Helsinki (2013 revision) and the Indian Council of Medical Research (ICMR) National Ethical Guidelines for Biomedical and Health Research Involving Human Participants (2017). Written informed consent was obtained from all participants prior to enrollment.

Sample size calculation

The sample size was estimated using the reported prevalence of vertebral fractures among patients with SpA (11.78%) from a previously published cohort [17], applying the formula \[ n = \frac{Z_{1-\alpha/2}^2 \times p(1-p)}{d^2} \] where Z_1−α/2_ = 1.96 (95% confidence level), p = 0.1178 (expected proportion of vertebral fracture), and d = 0.09 (absolute precision). This calculation yielded a minimum required sample size of 50 subjects. The relatively wide absolute precision of 9% was selected based on feasibility within the study period and resources available at a single tertiary care center. The authors acknowledge that this precision, combined with the expected low prevalence of vertebral fracture, was anticipated to yield a small absolute number of fracture events; accordingly, all analyses examining associations with vertebral fracture were pre-specified as exploratory and hypothesis-generating rather than confirmatory.

Participants

Adult patients (≥18 years) of either sex diagnosed with SpA according to the ESSG criteria [13], encompassing ankylosing spondylitis, ReA, PsA, enteropathic arthritis, juvenile-onset SpA, and undifferentiated SpA, who provided written informed consent were recruited using a consecutive sampling technique until the target sample size was reached. The ESSG criteria, rather than the contemporary ASAS classification criteria, were used for enrollment because they encompass a broader spectrum of the SpA phenotype, including peripheral-predominant, juvenile-onset, and enteropathic disease, without mandating advanced imaging (e.g., sacroiliac joint MRI) or HLA-B27 status as entry criteria, and were therefore considered more feasible and inclusive for a real-world cohort presenting to a general medicine department. The authors acknowledge that this choice limits direct comparability with cohorts classified using contemporary ASAS criteria.

The inclusion criteria comprised (i) adult patients (≥18 years) of either sex fulfilling ESSG criteria for SpA; (ii) patients willing to provide written informed consent; and (iii) patients attending the medicine OPD, IPD, or ICU during the study period. Exclusion criteria comprised patients with secondary causes of back pain (degenerative disc disease, malignancy, tuberculosis), metabolic bone disease unrelated to SpA, pregnancy or lactation, and those unwilling or unable to provide consent. Participants who withdrew consent, were lost to follow-up before completion of evaluation, or were subsequently found to have an alternative diagnosis were excluded from the final analysis.

Data collection

Data were collected using a predesigned, pretested case record form capturing demographic details, clinical history, physical examination findings, radiological results, and laboratory investigations. Each participant was assigned a unique study identification number to preserve confidentiality, with no personally identifiable information used in analysis. Data were transcribed into a Microsoft Excel spreadsheet (Microsoft Corp., Redmond, WA, USA), cross-checked by the principal investigator for completeness and accuracy, and cleaned prior to statistical analysis.

Procedure

Each participant underwent a structured clinical interview and comprehensive physical examination. Clinical history included demographic details, disease duration, pattern of joint involvement (axial versus peripheral), musculoskeletal features characteristic of SpA (enthesitis and dactylitis), extra-articular features (uveitis, psoriasis, inflammatory bowel disease, and nail changes), family history of SpA, comorbidities, and treatment history. Musculoskeletal examination assessed spinal mobility using the Modified Schober's test, chest expansion, occiput-to-wall distance, finger-to-floor distance, and cervical spine rotation.

All participants underwent plain radiographs of the pelvis (both sacroiliac joints) and spine (cervical, thoracic, and lumbar regions). Sacroiliitis was graded according to the Modified New York criteria [18]. Vertebral fracture was defined radiologically as a reduction in vertebral body height of ≥20% in the absence of significant trauma, assessed on lateral spine radiographs and graded semiquantitatively by percentage height loss, consistent with the principles of the Genant method (mild, 20%-25%; moderate, 25%-40%; severe, >40%), with MRI performed where radiographic findings were inconclusive. Laboratory investigations included complete blood count, erythrocyte sedimentation rate (ESR), C-reactive protein (CRP), renal and liver function tests, serum calcium, phosphorus and alkaline phosphatase, HLA-B27 typing, and rheumatoid factor/anti-cyclic citrullinated peptide antibodies to exclude rheumatoid arthritis. Data on bone mineral density, cumulative glucocorticoid exposure, smoking status, BMI, and prior non-vertebral fragility fractures were not systematically collected as part of the study protocol and could therefore not be evaluated as potential confounders of the vertebral fracture findings.

Outcome measures

The primary outcome was to describe the clinical, radiological, and biochemical profile of patients with SpA, including the prevalence and pattern of vertebral fractures. Secondary outcomes included assessment of disease activity using the BASDAI [14], a self-administered instrument comprising six 0-10 visual analogue scale (VAS) items assessing fatigue, spinal and peripheral joint pain, enthesitis-related tenderness, and the severity and duration of morning stiffness, with higher scores indicating greater disease activity; and the ASDAS-CRP [15], a composite index incorporating patient-reported back pain, duration of morning stiffness, patient global assessment, peripheral joint pain/swelling, and serum CRP, which stratifies patients into inactive (<1.3), moderate (1.3-2.1), high (2.1-3.5), and very high (>3.5) disease activity categories. Functional disability was assessed using the BASFI [16], a 10-item, 0-10 VAS-based questionnaire evaluating the ability to perform activities of daily living relevant to spinal and peripheral joint function, with higher scores indicating greater functional limitation. Although the BASDAI and BASFI were developed and validated primarily in ankylosing spondylitis/axial SpA, they were applied across the full cohort in this study given the substantial axial disease burden (82% axial-only or combined axial-peripheral involvement) and the absence of alternative instruments validated specifically for a mixed axial/peripheral Indian SpA population; this represents an interpretive caveat, particularly for patients with predominantly peripheral disease. These indices were analyzed for their correlation with the presence of vertebral fractures, sociodemographic variables, and inflammatory markers (ESR, CRP).

Statistical analysis

Data were analyzed using SPSS Statistics, version 26.0 (IBM Corp., Armonk, NY, USA). Continuous variables (age, BASDAI, ASDAS-CRP, BASFI, ESR, and CRP) were expressed as mean ± standard deviation or median (interquartile range) depending on distribution; categorical variables were expressed as frequencies and percentages. The chi-square test or Fisher's exact test was used to compare proportions between categorical variables, and the Student's t-test or Mann-Whitney U test was used to compare means of continuous variables between groups (e.g., presence versus absence of vertebral fracture). Fisher's exact test was applied in place of the Pearson chi-square test whenever more than 20% of cells in a contingency table had an expected count below 5; Fisher's exact test does not generate a distributional test statistic and is reported as an exact p-value. Pearson's correlation coefficient, or Spearman's rho, was used to assess correlations between continuous variables. A two-tailed p-value <0.05 was considered statistically significant. Missing data were handled by listwise exclusion. Given the small number of vertebral fracture events anticipated a priori, all statistical comparisons involving this outcome were considered exploratory, and results are presented as hypothesis-generating associations rather than confirmatory evidence of independent predictors or determinants.

## Results

Fifty patients meeting ESSG criteria for SpA were enrolled during the study period. The mean age of the cohort was 41.8 ± 10.9 years (median 42, IQR 34-49), with a marked male predominance (74%; male-to-female ratio 2.8:1). A higher proportion of patients were from rural areas (58%), and 64% belonged to lower-middle or upper-lower socioeconomic strata; manual laborers/farmers constituted the largest occupational group (36%) (Table 1).

**Table 1:** Sociodemographic characteristics of study participants (total n = 50) Mean ± SD age: 41.8 ± 10.9 years; median (IQR): 42 (34-49) years

Variable		N	%
Age group (years)	18-30	10	20.0
	31-40	16	32.0
	41-50	13	26.0
	51-60	8	16.0
	>60	3	6.0
Sex	Male	37	74.0
	Female	13	26.0
Residence	Rural	29	58.0
	Urban	21	42.0
Occupation	Manual laborer/Farmer	18	36.0
	Office worker/Clerk	9	18.0
	Homemaker	8	16.0
	Business/Self-employed	6	12.0
	Student	5	10.0
	Unemployed/Retired	4	8.0
Socioeconomic class (modified Kuppuswamy)	Lower middle	17	34.0
	Upper lower	15	30.0
	Upper middle	10	20.0
	Lower	6	12.0
	Upper	2	4.0

Disease duration ranged from less than one year to more than 10 years, with 28% of patients reporting symptoms for one to three years and 34% for more than five years. Ankylosing spondylitis was the most common subtype (54%), followed by psoriatic arthritis (16%) and undifferentiated SpA (12%) (Table 2).

**Table 2:** Disease duration and subtype distribution among study participants (total n = 50) SpA: Spondyloarthritis

Variable		N	%
Duration of disease (years)	<1	7	14.0
	1-3	14	28.0
	3-5	12	24.0
	5-10	11	22.0
	>10	6	12.0
Subtype of SpA	Ankylosing spondylitis	27	54.0
	Psoriatic arthritis	8	16.0
	Undifferentiated SpA	6	12.0
	Reactive arthritis	5	10.0
	Enteropathic arthritis	3	6.0
	Juvenile-onset SpA	1	2.0

Axial involvement alone was present in 56% of patients and combined axial-peripheral involvement in 26%. Extra-articular manifestations were observed in 64% of patients overall, most frequently enthesitis (38%), followed by uveitis (16%) and psoriasis (14%) (Table 3).

**Table 3:** Pattern of joint involvement and extra-articular manifestations (total n = 50) *Categories are not mutually exclusive; patients may have more than one manifestation

Variable		N	%
Pattern of joint involvement	Axial involvement only	28	56.0
	Combined axial and peripheral	13	26.0
	Peripheral involvement only	9	18.0
Extra-articular manifestation*	Enthesitis	19	38.0
	Uveitis	8	16.0
	Psoriasis	7	14.0
	Nail changes (pitting/onycholysis)	6	12.0
	Dactylitis	5	10.0
	Inflammatory bowel disease	3	6.0
	At least one manifestation	32	64.0

A positive family history of SpA was reported in 22% of patients. At least one comorbidity was present in 54%, most commonly hypertension (18%) and type 2 diabetes mellitus (14%). Nonsteroidal anti-inflammatory drugs (NSAIDs) were used by 94% of patients, physiotherapy/exercise therapy by 86%, conventional disease-modifying antirheumatic drugs (DMARDs) by 52%, and biologic agents by only 14% (Table 4).

**Table 4:** Family history, comorbidities, and treatment history (total n = 50) *Categories are not mutually exclusive. DMARDs: Disease-modifying antirheumatic drugs; NSAIDs: Nonsteroidal anti-inflammatory drugs; SpA: Spondyloarthritis

Variable		N	%
Family history of SpA	Present	11	22.0
	Absent	39	78.0
Comorbid condition*	Hypertension	9	18.0
	Type 2 diabetes mellitus	7	14.0
	Osteoporosis	6	12.0
	Dyslipidemia	5	10.0
	History of vertebral fracture	5	10.0
	Obesity (BMI ≥30 kg/m²)	4	8.0
	Others (e.g., hypothyroidism)	3	6.0
	At least one comorbidity	27	54.0
Treatment modality*	NSAIDs	47	94.0
	Physiotherapy/exercise therapy	43	86.0
	Analgesics/muscle relaxants	29	58.0
	Conventional DMARDs	26	52.0
	Corticosteroids (systemic/intra-articular)	12	24.0
	Biologic agents (e.g., TNF-α inhibitors)	7	14.0
	At least one DMARD or biologic	31	62.0

Spinal mobility parameters were markedly reduced (Table 5). The mean modified Schober's test was 3.2 ± 1.1 cm (below normal in 78% of patients), mean chest expansion was 2.8 ± 1.0 cm (reduced in 70%), and 82% of patients demonstrated restriction in two or more mobility parameters.

**Table 5:** Spinal mobility parameters among study participants (total n = 50)

Parameter	Mean ± SD	Normal range	Below normal (n)	%
Modified Schober's test (cm)	3.2 ± 1.1	≥5 cm	39	78.0
Chest expansion (cm)	2.8 ± 1.0	≥5 cm	35	70.0
Occiput-to-wall distance (cm)	5.6 ± 2.4	0 cm	31	62.0
Finger-to-floor distance (cm)	18.3 ± 7.5	≤10 cm	34	68.0
Cervical spine rotation (°)	48.5 ± 16.2	≥70°	28	56.0
Reduced mobility in ≥2 parameters	—	—	41	82.0

Radiologically, 54% of patients had Grade III-IV sacroiliitis on the Modified New York criteria, and vertebral fractures were identified in 12% of patients (predominantly wedge-type, 6%), most commonly involving the thoracic spine (50%), followed by the lumbar (33.3%) and cervical (16.7%) spine (Table 6).

**Table 6:** Radiological grading of sacroiliitis and vertebral fractures (total n = 50)

Variable		N	%
Sacroiliitis grade (Modified New York criteria)	Grade 0: Normal	5	10.0
	Grade I: Suspicious	7	14.0
	Grade II: Minimal abnormality	11	22.0
	Grade III: Moderate abnormality	17	34.0
	Grade IV: Severe abnormality (ankylosis)	10	20.0
Vertebral fracture type	Wedge fracture	3	6.0
	Biconcave fracture	2	4.0
	Crush fracture	1	2.0
	Total with vertebral fracture	6	12.0
	No vertebral fracture	44	88.0
Site of vertebral fracture (n = 6)	Thoracic spine	3	50.0
	Lumbar spine	2	33.3
	Cervical spine	1	16.7

Vertebral fracture prevalence increased progressively with disease duration, from 0% in patients with disease duration <3 years to 33.3% in those with disease duration >10 years. Because expected cell counts were <5 in several duration categories, this association was assessed with Fisher's exact test rather than the Pearson chi-square test, and was statistically significant (Fisher's exact test, p=0.018) (Table 7).

**Table 7:** Association between vertebral fracture and duration of disease (total n = 50) Fisher's exact test: p = 0.018 (used in place of the Pearson chi-square test because >20% of cells had an expected count <5); Fisher's exact test does not generate a distributional test statistic

Duration of disease	With fracture (n=6)	Without fracture (n=44)	Total
<3 years	0	21	21
Three to five years	1	11	12
Five to 10 years	3	8	11
>10 years	2	4	6
Total	6 (12.0%)	44 (88.0%)	50

The HLA-B27 positivity was observed in 80% of patients. Mean ESR was 37.2 ± 18.5 mm/hr (elevated in 62%), and mean CRP was 18.6 ± 10.4 mg/L (elevated in 68%); mild anemia was present in 36% of patients (Table 8). Patients with vertebral fractures had significantly higher mean ESR (51.8 ± 16.4 vs. 35.3 ± 17.9 mm/hr; independent-samples t-test, t(48)=2.14, p=0.038) and CRP (27.4 ± 9.8 vs. 17.3 ± 9.7 mg/L; t(48)=2.39, p=0.021) than patients without fractures (Table 9).

**Table 8:** Laboratory parameters and HLA-B27 status (total n = 50) ESR: Erythrocyte sedimentation rate, CRP: C-reactive protein

Parameter	Mean ± SD	Reference range	Abnormal (n)	%
Hemoglobin (g/dL)	12.4 ± 1.6	13-17 (M), 12-15 (F)	18	36.0
ESR (mm/hr)	37.2 ± 18.5	<20	31	62.0
CRP (mg/L)	18.6 ± 10.4	<6	34	68.0
Serum calcium (mg/dL)	8.9 ± 0.6	8.5-10.5	3	6.0
Alkaline phosphatase (IU/L)	119.4 ± 34.2	40-120	9	18.0
HLA-B27 positive	—	—	40	80.0

**Table 9:** Comparison of inflammatory markers between patients with and without vertebral fractures Independent-samples Student's t-test: ESR, t(48) = 2.14, p = 0.038; CRP, t(48) = 2.39, p = 0.021; ESR: Erythrocyte sedimentation rate; CRP: C-reactive protein

Parameter	With fracture (n=6)	Without fracture (n=44)	p-value
ESR (mm/hr)	51.8 ± 16.4	35.3 ± 17.9	0.038
CRP (mg/L)	27.4 ± 9.8	17.3 ± 9.7	0.021

Disease activity and functional indices demonstrated a predominantly active disease pattern (Table 10). Around 72% of patients had high disease activity on BASDAI (≥4.0; mean 4.8 ± 1.3), 74% had high or very high disease activity on ASDAS-CRP (>2.1), and 68% had moderate-to-severe functional limitation on BASFI (≥4.0).

**Table 10:** Disease activity (BASDAI, ASDAS-CRP) and functional status (BASFI) (total n = 50) Mean ± SD BASDAI: 4.8 ± 1.3; BASDAI: Bath Ankylosing Spondylitis Disease Activity Index; ASDAS-CRP: Ankylosing Spondylitis Disease Activity Score with C-reactive protein; BASFI: Bath Ankylosing Spondylitis Functional Index

Category		N	%
BASDAI	Low disease activity (<4.0)	14	28.0
	High disease activity (≥4.0)	36	72.0
ASDAS-CRP	Inactive disease (<1.3)	3	6.0
	Moderate disease activity (1.3-2.1)	10	20.0
	High disease activity (2.1-3.5)	24	48.0
	Very high disease activity (>3.5)	13	26.0
BASFI	Mild limitation (<4.0)	16	32.0
	Moderate limitation (4.0-6.0)	22	44.0
	Severe limitation (>6.0)	12	24.0

## Discussion

This cross-sectional study of 50 patients with SpA from a tertiary care hospital in Central India demonstrates a disease profile broadly consistent with global epidemiological patterns, while also highlighting features specific to a predominantly rural, socioeconomically disadvantaged population. The mean age of 41.8 ± 10.9 years and the concentration of patients in the 31 to 50 years age band are consistent with the well-described onset of axial SpA in young to middle adulthood, typically before the age of 45 years [3,6]. The marked male predominance (74%; ratio 2.8:1) mirrors the classical epidemiology of ankylosing spondylitis, although contemporary registries that include non-radiographic disease report a narrowing sex gap, partly attributable to diagnostic delay and under-recognition of SpA in women [6,11]. The predominance of rural residents (58%) and patients from lower-middle/upper-lower socioeconomic strata (64%) in this cohort is consistent with community-based Indian data suggesting that SpA is not confined to urban populations and with multinational analyses linking lower socioeconomic status to higher disease activity and greater disability, independent of access to biologic therapy [19-21]. The predominance of manual laborers/farmers (36%) further suggests that physically demanding occupations may compound symptom burden and functional impact in this setting, in line with evidence that axial SpA substantially affects work participation and productivity [10]. Ankylosing spondylitis was the most frequent subtype (54%), followed by psoriatic arthritis (16%) and undifferentiated SpA (12%), reflecting the recognized clinical heterogeneity of the SpA spectrum [1,6]. Enteropathic arthritis was identified in 6% of the cohort, consistent with the well-described association between inflammatory bowel disease and the SpA spectrum, in which axial and/or peripheral joint involvement has been reported in a substantial proportion of patients with underlying bowel inflammation [22]. Axial disease predominated (82% when axial-only and combined phenotypes are considered), consistent with axSpA being the primary clinical phenotype encountered in rheumatology and general medicine referral settings [4,5]. Peripheral musculoskeletal features of SpA-enthesitis (38%) and dactylitis (10%) were common, while true extra-articular manifestations, most frequently uveitis (16%) and psoriasis (14%), were also frequent, corroborating the systemic, multi-organ nature of SpA described in earlier meta-analyses [12].

A positive family history was noted in 22% of patients, and HLA-B27 positivity was found in 80%, reaffirming the strong genetic and heritable basis of SpA that is predominantly, though not exclusively, mediated by HLA-B27 [7]. More than half of the cohort (54%) had at least one comorbidity, most commonly hypertension and type 2 diabetes mellitus, reflecting the known association between chronic systemic inflammation and cardiometabolic risk in SpA populations [10]. Therapeutically, NSAIDs (94%) and physiotherapy (86%) were the dominant treatment modalities, consistent with their established first-line role in axSpA management [23,24], while biologic agents were used in only 14% of patients, considerably lower than utilization rates reported in high-income settings, likely reflecting cost and accessibility constraints typical of resource-limited healthcare systems.

Spinal mobility was markedly impaired, with 82% of patients showing restriction in two or more parameters and a mean modified Schober's test of 3.2 ± 1.1 cm, comparable to values reported in other AS cohorts using the same or related metrology indices [25,26]. Radiologically, 54% of patients had grade III-IV sacroiliitis, indicating that a majority presented with established structural damage, likely reflecting diagnostic delay and limited early access to imaging, a pattern also observed in other Indian cohorts [26].

Vertebral fractures were identified in 12% of patients (n=6), a prevalence broadly consistent with the 11.8% reported by Geusens et al. in a European cohort that also informed the sample size calculation for this study [17]; given the small absolute number of fracture events, these findings should be interpreted as exploratory and hypothesis-generating rather than definitive. Wedge-type fractures predominated, and the thoracic spine was the most frequently affected site (50%), consistent with the recognized vulnerability of the thoracolumbar junction in ankylosed, osteoporotic spines [27-29]. Fracture prevalence was numerically higher with longer disease duration, rising from 0% in patients with less than three years of disease to 33.3% in those with more than 10 years (Fisher's exact test, p=0.018); patients with vertebral fractures also had numerically higher ESR (t(48)=2.14, p=0.038) and CRP levels (t(48)=2.39, p=0.021) than those without fractures. These associations are consistent with the broader concept that persistent systemic inflammation, cumulative disease duration, and progressive ankylosis jointly contribute to skeletal fragility in SpA [17,27,30,31]. However, with only six fracture events, this study was not adequately powered to adjust for other established risk factors for vertebral fracture, including age, bone mineral density, cumulative glucocorticoid exposure, smoking, BMI, and prior non-vertebral fractures, none of which were systematically assessed in this cohort. The observed associations with disease duration and inflammatory markers should therefore be regarded as exploratory signals warranting confirmation in larger, prospective, multivariable studies rather than evidence of independent or causal predictors.

Disease activity indices confirmed a predominantly active disease pattern: 72% of patients had high BASDAI scores, and 74% had high or very high ASDAS-CRP scores, while 68% demonstrated moderate-to-severe functional limitation on BASFI. This concordance between subjective and composite disease activity measures and functional outcome is consistent with prior validation studies of these indices [14-16]. As BASDAI and BASFI were developed and validated primarily in axial SpA/ankylosing spondylitis, their application across this heterogeneous cohort, which included patients with predominantly peripheral disease, should be interpreted with some caution, and disease-activity findings may not be equally generalizable across all SpA subtypes. Nonetheless, these findings underscore recommendations advocating a treat-to-target approach, with early and sustained control of inflammation to prevent structural progression and preserve function [23,31].

Strengths

This study has several notable strengths. It employed a structured, predesigned data collection protocol incorporating validated, internationally recognized disease activity and functional indices (BASDAI, ASDAS-CRP, BASFI) alongside standardized clinical and radiological assessment of sacroiliitis and vertebral fracture, allowing systematic characterization of a real-world SpA cohort from an Indian tertiary care setting where such comprehensive data remain scarce. The inclusion of a broad range of SpA subtypes, sociodemographic variables, and comorbidities provides a holistic clinical picture that may be of practical relevance to clinicians managing SpA in similar resource-limited settings. The study also explicitly incorporated vertebral fracture as a clinically important, though frequently overlooked, structural outcome, and applied Fisher's exact test rather than the Pearson chi-square test where expected cell counts were small, reflecting appropriate statistical handling of a rare outcome rather than overstating the precision of these estimates.

Limitations

This study's cross-sectional design precludes causal inference regarding the temporal relationship between inflammation, functional impairment, and radiographic progression. The relatively small sample size (n=50) and single-center, tertiary-care setting may limit the generalizability of the findings and may be subject to referral or selection bias toward more severe disease phenotypes. The use of ESSG rather than contemporary ASAS classification criteria may limit direct comparability with more recently published axial SpA cohorts. The heterogeneity of included SpA subtypes, combined with the application of BASDAI and BASFI instruments developed primarily for axial disease across the full cohort, represents an additional source of interpretive uncertainty. Most critically, only six patients had vertebral fractures; this very small number of events substantially limits the statistical power, precision, and generalizability of all fracture-related analyses, which should be regarded as exploratory and hypothesis-generating rather than confirmatory. Established risk factors for vertebral fracture, including bone mineral density, cumulative glucocorticoid exposure, smoking status, BMI, and prior non-vertebral fractures, were not systematically assessed and could not be adjusted for as potential confounders. Reliance on self-reported indices (BASDAI, BASFI) may introduce subjective bias, and resource constraints precluded bone mineral density testing or dual-energy X-ray absorptiometry in all participants, potentially underestimating early osteoporotic changes. Variation in treatment exposure, including limited biologic therapy access, may also have confounded disease activity and outcome profiles.

## Conclusions

In this cross-sectional cohort, SpA predominantly affected young to middle-aged men from rural and lower socioeconomic backgrounds, with a high burden of active inflammatory disease, restricted spinal mobility, and structural damage. Vertebral fractures, although present in only a small subset of patients, were more frequent with longer disease duration and higher inflammatory markers in this exploratory analysis. These findings highlight the importance of early diagnosis, routine monitoring using standardized disease activity and functional indices, and clinical vigilance for vertebral fracture as an under-recognized structural complication of SpA, particularly in resource-limited settings where diagnostic and therapeutic delays remain common. Larger, multicentric, and longitudinal studies incorporating bone mineral density assessment and other established fracture risk factors are warranted to validate these exploratory findings and to better define predictors of structural and functional progression in this population.
